# Trends in Antihypertensive Medicine Utilization in the Republic of Srpska, Bosnia and Herzegovina: An Eleven-Year Follow-Up

**DOI:** 10.3389/fphar.2022.889047

**Published:** 2022-06-15

**Authors:** Dragan Kalinić, Ranko Škrbić, Duško Vulić, Nataša Stojaković, Svjetlana Stoisavljević-Šatara, Miloš P. Stojiljković, Vanda Marković-Peković, Ana Golić Jelić, Nataša Pilipović-Broćeta, Nathan D. Wong, Brian Godman

**Affiliations:** ^1^ Centre for Biomedical Research, Faculty of Medicine, University of Banja Luka, Banja Luka, Bosnia and Herzegovina; ^2^ Department of Pharmacology, Toxicology and Clinical Pharmacology, Faculty of Medicine, University of Banja Luka, Banja Luka, Bosnia and Herzegovina; ^3^ Centre for Specializations and Continuous Medical Education, Faculty of Medicine, University of Banja Luka, Banja Luka, Bosnia and Herzegovina; ^4^ Department of Pharmacy, Faculty of Medicine, University of Banja Luka, Banja Luka, Bosnia and Herzegovina; ^5^ Family Medicine Teaching Center, Primary Health Care of Banja Luka, Banja Luka, Bosnia and Herzegovina; ^6^ Heart Disease Prevention Program, Division of Cardiology, University of California, Irvine, Irvine, CA, United States; ^7^ Institute of Pharmacy and Biomedical Sciences, University of Strathclyde, Glasgow, United Kingdom; ^8^ Centre of Medical and Bio-allied Health Sciences Research, Ajman University, Ajman, United Arab Emirates; ^9^ Division of Public Health Pharmacy and Management, School of Pharmacy, Sefako Makgatho Health Sciences University, Pretoria, South Africa

**Keywords:** antihypertensive medicines, medicine utilisation, ATC/DDD analyses, national guidelines, prescribing practices

## Abstract

**Background:** In last 2 decades, there have been substantial changes in the utilization patterns of antihypertensive medicines following new clinical trials and the introduction of new treatment guidelines. The aim of this study was to analyze utilization and prescribing patterns regarding antihypertensive medicines in the Republic of Srpska, Bosnia and Herzegovina during an 11-years follow-up according to national and European treatment guidelines.

**Methods:** In this retrospective, observational study, medicine utilization data were analyzed between 2009–2019 period using the ATC/DDD methodology and expressed as the number of DDD/1,000 inhabitants/day (DID/TID). The medicine utilization 90% (DU90%) method was used for determine the quality of prescribing.

**Results:** During the observed period, the use of antihypertensive medicines increased more than 3-times (125.97 DDD/TID in 2009 vs 414.95 DDD/TID in 2019), corresponding to a rise in the prevalence of hypertensive patients from 91.7/1,000 to 186.3/1,000 in the same period. This was mainly driven by increased use of angiotensin converting enzyme inhibitors with 241.69%, beta blockers with 146.87%, calcium channel blockers with 251.55%, and diuretics with 178.95%. Angiotensin receptor blockers were the fastest growing group of antihypertensive medicines in this period and their utilization increased nearly 40 times.

**Conclusions:** The overall antihypertensive medicines utilization was largely influenced by national and ESH/ESC guidelines and strongly corresponded to the positive medicine list of the national health insurance fund. Antihypertensive medicines utilization is comparable with medicine utilization trends in other countries.

## Introduction

Hypertension is the most common chronic disease of modern era and one of the leading causes of cardiovascular morbidity and mortality accounting for 10.4 million deaths globally per year (Global Health Metrics, 2018; Stanaway et al., 2018). In 2010, an estimated 1.39 billion people globally had hypertension (Mills et al., 2020). More recently, it is estimated that 1.28 billion people aged between 30 and 79 years have hypertension globally, greater in men than women, with an appreciable number unaware of their condition (NCD Risk Factor Collaboration (NCD-RisC), 2021; World Health Organisation, 2021a). Morbidity trends showed a clear shift away from high-income countries (HIC) to low and middle-income countries (LMIC) (NCD Risk Factor Collaboration (NCD-RisC), 2021; World Health Organisation, 2021a), with over a billion people with hypertension now living in LMICs (Mills et al., 2020). The most recent estimates suggest that, across both the European Union and non-European Union countries, the prevalence of hypertension is around 40% and tends to be higher in Central and Eastern European countries than other European countries (NCD Risk Factor Collaboration (NCD-RisC), 2021). This is reflected in the aged-standardized prevalence of hypertension among European countries varying for instance from 20.8% in Spain, 24.4% in France, 24.6% in Sweden, 25.0% in Germany, 26.1% in Belgium, and 26.2% in Greece to 38.9% in Latvia, 41.3% in Bosnia and Herzegovina, 42.7% in Poland and 45.3% in Croatia (NCD Risk Factor Collaboration (NCD-RisC), 2021; World Health Organization, 2019).

Several factors impact on prevalence rates. These include smoking rates, current lifestyles, income and education with some of these captured as part of human development indices (Tirapani and Fernandes, 2019; Zeng et al., 2020). Due to the growing global burden of cardiovascular diseases, international professional bodies, as well as the national health authorities in many countries, have developed and disseminated treatment guidelines in order to improve the management of hypertensive patients. This includes the World Health Organization (WHO), the International Society of Hypertension and those from European societies (Williams et al., 2018; Unger et al., 2020; Verdecchia et al., 2020; World Health Organisation, 2021b). The trends in the utilization of different medicines for the management of hypertension can provide important insight into how fast the therapeutic recommendations are being adopted in clinical practice and the impact of different therapeutic options on treatment outcomes. However, in both LMICs and HICs the ambiguities of the latest guidelines can be met with confusion among healthcare professionals, anxiety among patients, and the fear among healthcare providers that new treatments will additionally increase treatment costs (Rehan et al., 2017; Messerli and Bangalore, 2018; Poulter et al., 2018). This includes issues whether diuretics, calcium channel blockers (CCBs), diuretics combined with angiotensin-converting enzyme inhibitors (ACEIs) or with CCBs should be first line, and whether treatment approaches need to be adapted bases on age and ethnicity (Kaiser et al., 2014; Mbui et al., 2017; Benetos et al., 2019; Mashozhera et al., 2021). However, costs of medicines to treat hypertension and a number of other cardiovascular diseases are now less of an issue across Europe with these typically available as low-cost generics (Markovic-Pekovic et al., 2012), e.g., the prices of generic losartan were already 88% lower shortly after their availability in Scotland with similar low prices in Sweden, and prices of generic statins have been as low as 2%–4% of pre-patent loss prices among European countries (Woerkom et al., 2012; Bennie et al., 2013; Godman et al., 2013; Leporowski et al., 2018). The effectiveness of major antihypertensive treatment options including diuretics, CCBs, ACEIs, ARBs, and beta blockers (BBs) are well documented (Neal et al., 2000; Psaty et al., 2003; Mancia et al., 2013).

However, we are aware that the treatment of hypertension differs among European countries and between Europe and other geographic regions despite international and Pan-European guidance. Comparative data suggests that the use of antihypertensive treatment is lower in Europe (England 25%, Germany 26%, Spain 27%, and Italy 32%) than in the United States (53%) and Canada (36%) (Wolf-Maier et al., 2004). As mentioned, despite improvements in the availability of different antihypertensive medications with different side-effect profiles, an appreciable number of patients with arterial hypertension remain uncontrolled while on treatment (NCD Risk Factor Collaboration (NCD-RisC), 2021; World Health Organisation, 2021a). The European Study on Cardiovascular Risk Prevention and Management in Usual Daily Practice (EURIKA) published in 2016 documented a 51.6% prevalence of uncontrolled hypertension in Europe, ranging from 38.6% in Greece to 59.7% in Turkey (Borghi et al., 2016).

In 2003, the European Society of Hypertension (ESH) and the European Society of Cardiology (ESC) jointly published guidelines for hypertension treatment that replaced the previous guidelines issued by the WHO and the International Society of Hypertension (ISH) (Zanchetti et al., 1993; Zanchetti, 20032003), which have subsequently been updated. A year later, the Cardiology Society of the Republic of Srpska published the first national guideline for hypertension in collaboration with the Ministry of Health and Social Welfare, and it became the leading guideline for hypertension used in daily practice among family physicians (Guidelines Committee, 2004; Pilipovic-Broceta et al., 2018). Furthermore, these guidelines were updated in 2009 and 2015 (Guidelines Committee, 2009; Ministry of Health and Social Welfare of Republic of Srpska, 2015), and together with the guidelines for other cardiovascular diseases, have significantly influenced medicine utilization patterns (Markovic-Pekovic et al., 2009; Marković-Peković et al., 2016).

Consequently, the aim of this study was firstly, to examine the 11-year trends in the prescribing of antihypertensive medicine in the Republic of Srpska. Secondly, to analyze the most prescribed classes of hypertensive medicines and the trends in their usage. Thirdly, the compatibility of the usage patterns seen versus national and European treatment guidelines. This is because adherence to guidelines is seen as a robust quality indicator with increased adherence to guidelines improving patient outcomes (Asmar et al., 2007; Mbui et al., 2017; NICE, 2017; Kamath et al., 2020; Mashozhera et al., 2021). We have shown in an earlier publication when researching medicines for hypertension prescribed among polypharmacy patients that prescribing appeared to compliment guideline recommendations; however, this was not formalized (Marković-Peković et al., 2016). Consequently, there was a need for this separate study. The findings can help to inform future activities in the Republic of Srpska and wider.

## Materials and Methods

A retrospective, observational study was conducted to analyze the utilization of antihypertensive medicines of Republic of Srpska during the period 2009–2019. The Republic of Srpska is one of two constitutive entities of Bosnia & Herzegovina with a total population of 1.2 million (Republic of Srpska Institute of Statistics, 2020). It has executive and legislative functional responsibilities covering healthcare policies. The Ministry of Health and Social Welfare is responsible for planning, regulation and management functions, while the Health Insurance Fund (HIF) provides compulsory health insurance coverage for the entire population based on solidarity and mutuality (Markovic-Pekovic et al., 2012). All inhabitants in the Republic have equal and free access to universal health care, including the services at primary, secondary and tertiary health care levels provided in all health institutions, which have a contract with the HIF. Most of the services, including prescribed medicines, are reimbursed by the HIF. The list of reimbursed medicines, the so-called “positive list”, is based on Anatomical Therapeutic Chemical (ATC) classification and comprises medicine listed by international non-proprietary names (INN-ATC level 5) recommended by the guidelines of clinical practice (Markovic-Pekovic et al., 2012). The positive list is updated regularly, at least once a year, and published in the Official Gazette. The vast majority of antihypertensive medicines are included in the positive medicines list and prescribed by doctors at the primary healthcare level. These prescribed medicines are dispensed in community pharmacies as these are prescription medicines only. Medicines proposed by physicians at discharge from hospitals or at ambulatory care visits are also dispensed from community pharmacies and consequently are included in primary healthcare sector sales (Health Insurance Fund of Republic of Srpska, 2021a).

The medicines utilization data were retrieved from Public Health Institute (PHI) of the Republic of Srpska. The PHI is responsible for collecting medicines utilization data from all health institutions and preparing an annual report (Markovic-Pekovic et al., 2012). Medicines utilization analysis was undertaken using the ATC/DDD (Defined Daily Dose) methodology (World Health Organization, 2003), which is the internationally accepted methodology for measuring medicines utilization within and across populations (Godman et al., 2014a; Godman et al., 2014b; Moon et al., 2014). DDDs are defined as the amount of medicines most commonly used for the most common indication in adults. It is a suitable measure to describe and compare medicines utilization patterns between different geographical regions and health facilities (Godman et al., 2014a; Godman et al., 2014b; Moon et al., 2014; Marković-Peković et al., 2016). Data on outpatient medicines utilization are typically expressed in DDDs/1,000 inhabitants/day (DIDs/TID) for comparative purposes (World Health Organization, 2003; Vončina et al., 2011; Godman et al., 2014a). For assessing the quality of antihypertensive prescribing, the medicines utilization 90% (DU90%) parameter was used. DU90% reflects the number of medicines that account for 90% of medicine prescriptions and serve as a general quality indicator (Bergman et al., 1998; Gustafsson et al., 2011; Dhananjay et al., 2016). Changes in prescribing patterns are measured with the index of change, a statistical measure of changes in a representative group of individual data points.

The antihypertensive treatment recommendations were followed in line with the updated National Guidelines (2009 and 2015 Guidelines for arterial hypertension of Republic of Srpska (Guidelines Committee, 2009; Ministry of Health and Social Welfare of Republic of Srpska, 2015)), the Guidelines of the ESC (2018 Practice Guidelines for the Management of Arterial Hypertension of the ESH and the ESC (Williams et al., 2018)), and the actual prescription patterns were compared to similar data from published sources.

The number of patients with hypertension (I10-I25 code according to the WHO International Classification of Diseases–ICD-revision 10) was obtained from the HIF of the Republic of Srpska data information system. The data extraction was carried out anonymously, with patients fully de-identified and thus this investigation was exempt from review by the institutional review board. This is in line with previous publications for the Republic of Srpska (Markovic-Pekovic et al., 2009; Markovic-Pekovic et al., 2012; Marković-Peković et al., 2016).

Changes in prescribing patterns were presented with the index of change, a statistical measure of changes in a representative group reflecting the changes in prescribing in DDDs/1,000 in 2019 compared with 2009.

## Results

During the 11-year period, there was a continual increase in the number of hypertensive patients in the Republic of Srpska. In 2009, the number of these patients was 108,051 with a prevalence of 91.7/1,000 people and in 2019 that number almost doubled with 212,874 patients and a prevalence of 186.3/1,000 people (Table 1).

**TABLE 1:** Percent of patients over 65 years, and total number and prevalence of patients with hypertension in the Republic of Srpska during the period 2009–2019.

Years	2009	2010	2011	2012	2013	2014	2015	2016	2017	2018	2019
Percent (%) of patients ≥65 years	51.43	51.02	52.52	51.46	51.85	52.03	53.14	53.51	54.77	55.6	57.36
Total number of hypertensive patients	108051	115615	123708	132367	141633	151548	171146	184168	193910	201412	212874
Prevalence of hypertension (‰)	91.7	98.3	105.3	112.8	120.9	129.9	147.3	159.1	168.2	175.5	186.3

**FIGURE 1:**
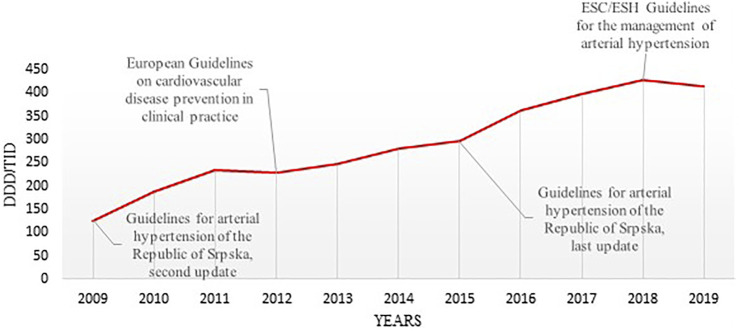
The timeline of utilization of antihypertensive medicines in the Republic of Srpska and publication years of the most relevant international and national therapeutic guidelines (DDD–Defined Daily Dose; TID–Thousand Inhabitant per Day; ESC–the European Society of Cardiology ESH–the European Society of Hypertension).

**FIGURE 2:**
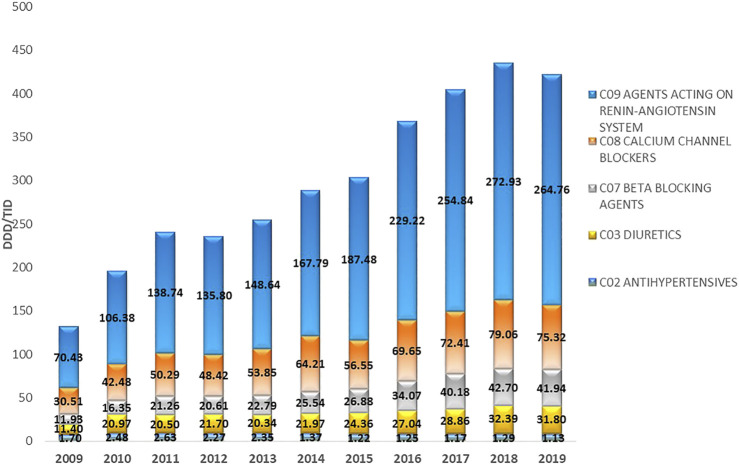
The utilization of major antihypertensive medicine groups in the Republic of Srpska expressed as DDD/TID during the period 2009–2019. DDD–Defined Daily Dose; TID–Thousand Inhabitant per Day.

Total medicine consumption also significantly increased during this period from 448.16 DDDs/TID in 2009 to 1,157.7 DDDs/TID in 2019. Out of this, the cardiovascular medicines (group C) were the most prescribed group of medicines accounting for 36.6%–42.04% of all medicines dispensed during this period. Among these, antihypertensive medicine was the most prescribed, with their total utilization increasing more than 3-fold from 125.98 DDDs/TID in 2009 to 414.95 DDDs/TID in 2019 (Table 2; Figures 1, 2).

**TABLE 2:** The utilization of cardiovascular medicines (group C) and antihypertensive medicines expressed in DDD/TID and as a share (%) of the total medicine utilization in the Republic of Srpska during the period 2009–2019.

Year	2009	2010	2011	2012	2013	2014	2015	2016	2017	2018	2019
Overall medicine use in DDD/TID	448.16	622.31	746.57	731.55	764.28	841.68	861.23	969.54	1,036.31	1,225.86	1,157.70
Group C DDD/TID	163.81	242.89	294.4	285.39	300.85	338.72	348.74	422.86	460.17	502.43	486.93
%	36.55	39.03	39.43	39.01	39.36	40.24	40.49	43.61	44.40	41.00	42.10
AHD DDD/TID	125.98	188.65	233.42	228.79	247.97	280.88	296.38	361.54	397.46	428.36	414.95
%	28.11	30.31	31.27	31.27	32.44	33.37	34.41	37.29	38.35	34.94	35.84

DDD, Defined Daily Dose; TID, Thousand Inhabitants per Day; AHD, Antihypertensive medicines according to ATC, classification.

The most prescribed antihypertensive medicine class during this period belonged to ACEIs (C09), with their utilization increasing from 69.78 DDDs/TID in 2009 to 238.43 DDDs/TID in 2019, equivalent to an increase of 241.69%. This was followed by CCBs (C08), where their utilization continuously increased from 30.51 DDDs/TID in 2009, to 75.32 DDDs/TID in 2019, equivalent to 146.87%. The utilization of BBs (C07) increased from 11.93 DDDs/TID in 2009 to 41.94 DDDs/TID in 2019, an increase of 251.55%. The fastest growing subgroup of antihypertensive medicines were ARBs with an approximately 40-fold increase over the study period. This compared with the subgroup antihypertensives (C02), which comprises different types of antihypertensives including central and peripheral acting antiadrenergic agents, agents acting on arterial smooth muscles, and their combinations with diuretics and alkaloids, which showed a decrease of –33.53% (Table 2; Figure 2).

**FIGURE 3:**
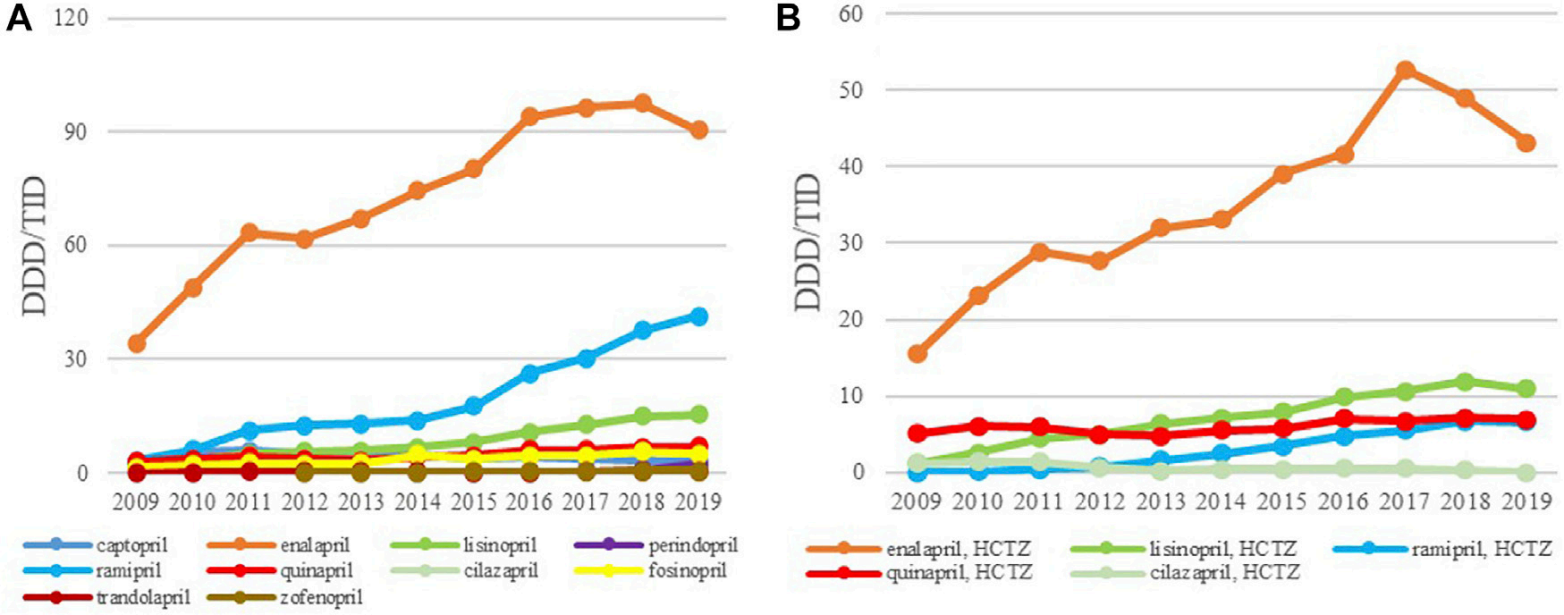
The utilization of ACEI as monotherapy **(A)** and ACEI in combination with diuretics **(B)**, expressed as DDD/TID in the Republic of Srpska from 2009 to 2019 (ACEI–Angiotensin Converting Enzyme Inhibitros; DDD–Defined Daily Dose; TID–Thousand Inhabitant per Day).

**FIGURE 4:**
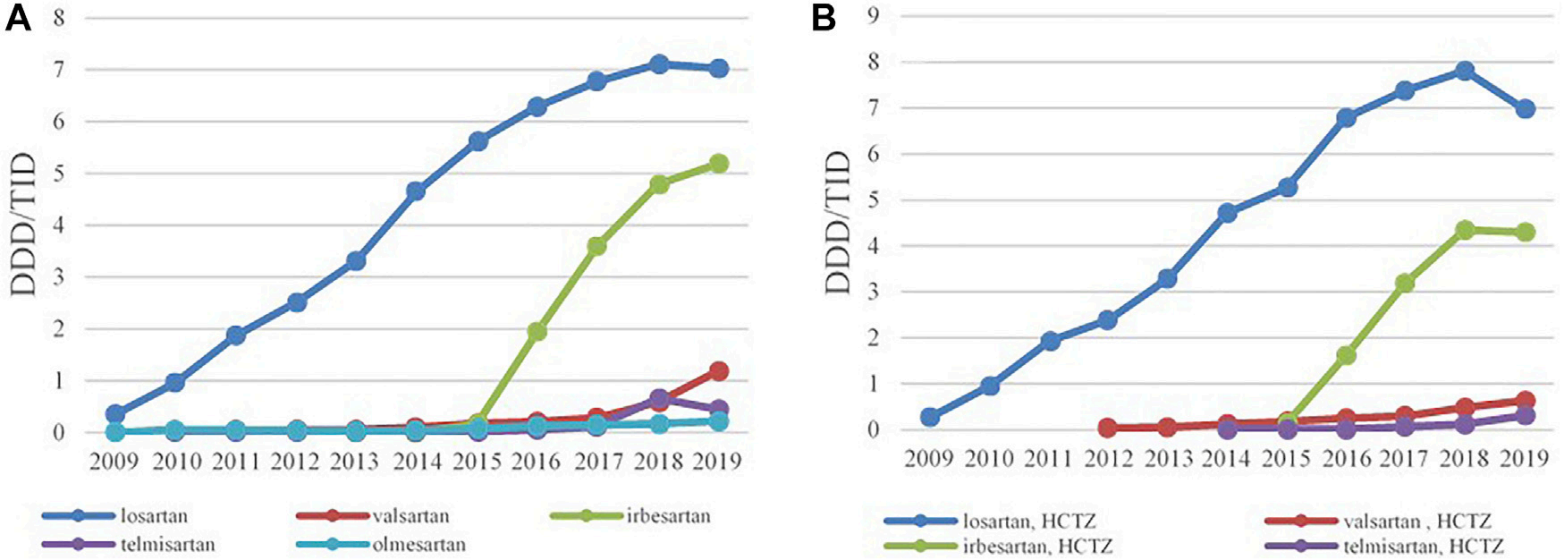
The utilization of ARBs as monotherapy **(A)** and ARBs in combination with HCTZ **(B)**, expressed as DDD/TID in the Republic of Srpska from 2009 to 2019 (ARB–Angiotensin Receptor Blockers; HCTZ–Hydrochlorothiazide; DDD–Defined Daily Dose; TID–Thousand Inhabitant per Day).

The ACEIs as monotherapy (C09A) were the leading most-prescribed subgroup with 46.67 DDDs/TID in 2009 rising to 165.12 DDDs/TID in 2019. The utilization of ACEIs in combination with diuretics, mainly with hydrochlorothiazide (HCTZ), increased by 217.22% in the observed period (Table 3; Figure 3A,B). Among the agents acting on the renin-angiotensin system (C09), the highest growth rates were in the ARB subgroup (C09D); from 0.28 DDDs/TID in 2009 to 12.23 DDDs/TID in 2019 (Table 3; Figure 4).

**TABLE 3:** The utilization of antihypertensive medicine groups and subgroups expressed as DDD/TID in the Republic of Srpska during the period 2009–2019.

ATC	Pharmacological Groups and Subgroups	2009	2010	2011	2012	2013	2014	2015	2016	2017	2018	2019
		DDDs/TID										
C09A	ACEI	46.67	70.89	93.68	91.55	96.76	109.28	118.74	147.16	154.80	167.65	165.12
C09B	ACEI + HCTZ	23.11	33.49	41.13	39.18	45.09	48.82	56.94	64.73	78.17	79.16	73.31
C08	CCB	30.51	42.48	50.29	48.42	53.85	64.21	56.55	69.95	72.41	79.06	75.32
C07	BB	11.93	16.35	21.26	20.61	22.79	25.54	26.88	34.07	40.18	42.70	41.94
C03	DU	11.40	20.97	20.50	21.70	20.34	21.97	24.36	27.04	28.86	32.39	31.80
C09C	ARB	0.37	1.04	1.98	2.62	3.44	4.82	6.08	8.66	10.94	13.33	14.10
C09D	ARB + HCTZ	0.28	0.95	1.94	2.44	3.35	4.85	5.61	8.68	10.93	12.78	12.23
C02	AH	1.70	2.48	2.63	2.27	2.35	1.37	1.22	1.25	1.17	1.29	1.13
Total DDDs/TID		125.97	188.65	233.41	228.79	247.97	280.86	296.38	361.54	397.46	428.36	414.95

ACEI, Angiotensin Converting Enzyme Inhibitors; CCB, Calcium Channel Blockers; BB, Beta Blockers; DU, Diuretics; ARB, Angiotensin Receptor Blockers; HCTZ, hydrochlorothiazide; AH, antihypertensives.

Overall, enalapril was the most-prescribed ACEI during the 11-year follow-up period with 34.27 DDDs/TID in 2009, reaching the 90.37 DDDs/TID in 2019, an increase of 163.7%. Ramipril was the second most prescribed medicine in this subgroup with its utilization increasing more than 13 times, from 3.11 DDDs/TID in 2009 to 41.44 DDDs/TID in 2019 (Figure 3A), while lisinopril was the third most prescribed ACE inhibitor in 2019 with 15.34 DDDs/TID. Among the fixed combination of ACEI and diuretics, the greatest utilization belonged to the enalapril-HCTZ combination, with 15.52 DDDs/TID in 2009 and 43.20 DDDs/TID in 2019, showing the increase rate of 178.35%, followed by the lisinopril-HCTZ combination with 10.98 DDDs/TID in 2019 (Figure 3B).

Losartan was the leading ARB, both as monocomponent medicine as well as in combination with HCTZ, and its utilization has increased constantly from 0.35 DDDs/TID and 0.28 DDDs/TID in 2009 to 7.03 DDDs/TID and 6.98 DDDs/TID in 2019, respectively. Since 2015, irbesartan has become the second most prescribed ARB, both as monotherapy and in combination with HCTZ, followed by valsartan and telmisartan (Figure 4A,B).

**FIGURE 5:**
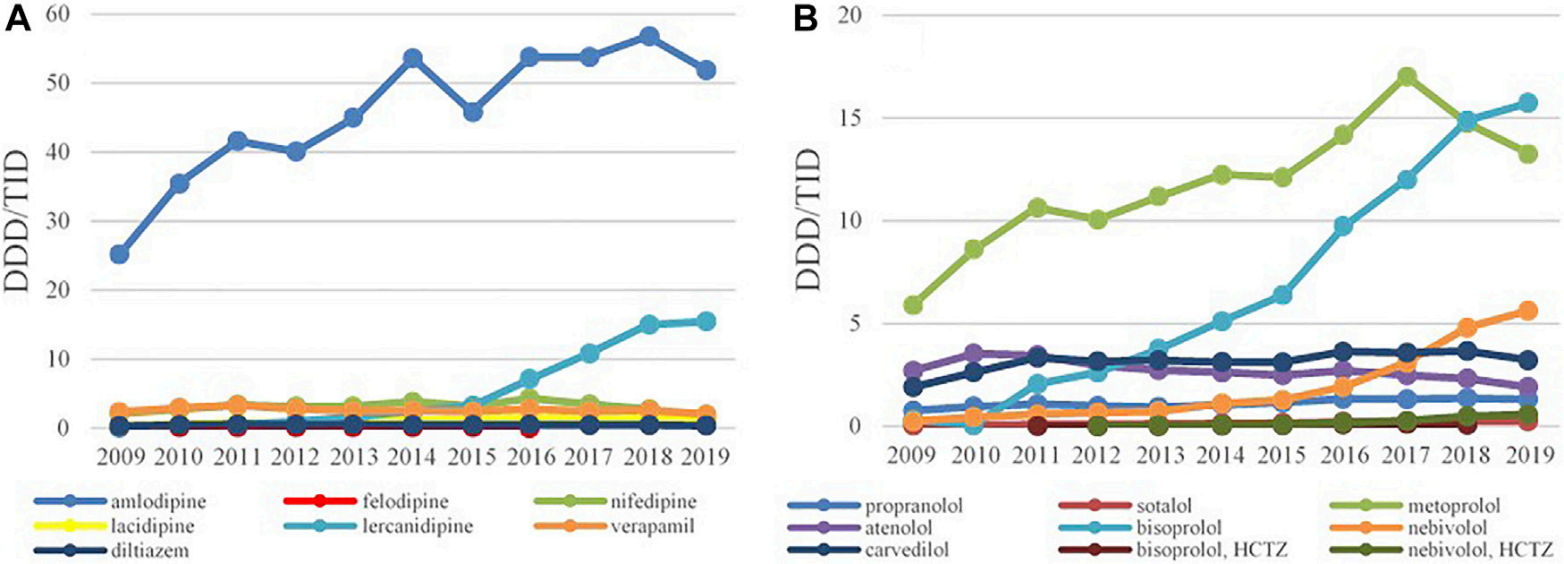
The utilization of CCB **(A)** and BB **(B)**, expressed as DDD/TID in the Republic of Srpska from 2009 to 2019 (CCB–Calcium Channel Blockers; BB–Beta Blockers; DDD–Defined Daily Dose; TID–Thousand Inhabitant per Day).

Amlodipine was the most prescribed CCBs with 25.21 DDDs/TID in 2009 and 51.92 DDDs/TID in 2019, with the increase rate of 105.95%. The second most prescribed CCB was lercanidipine whose consumption has started to increase significantly from 2013 (Figure 5A). The prescribing pattern of BBs have appreciably changed during the study period. Until 2017, metoprolol was the most-prescribed BBs reaching 17.3 DDDs/TID, but in a following 2 years the utilization of metoprolol started to decline, while bisoprolol became the leading BBs with 15.74 DDDs/TID in 2019, followed by nebivolol with 5.63 DDDs/TID (Figure 5B).

**FIGURE 6:**
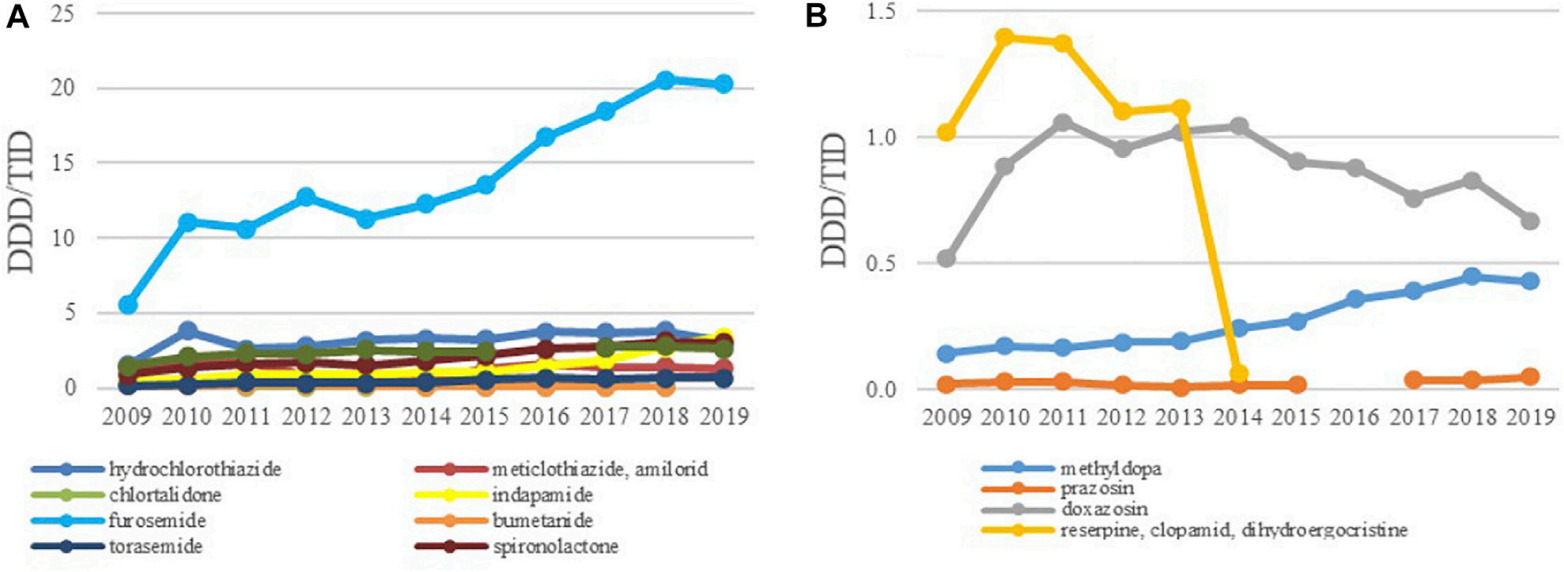
The utilization of diuretics **(A)** and ‘antihypertensives’ **(B)**, expressed as DDD/TID in the Republic of Srpska from 2009 to 2019 (DDD–Defined Daily Dose; TID–Thousand Inhabitant per Day).

Among the diuretics, furosemide had the biggest growth rate of 267.1%; its utilization increased from 5.53 DDDs/TID in 2009 to 20.30 DDDs/TID in 2019 (Figure 6A). The use of other diuretics such as HCTZ, spironolactone, indapamide and torasemide increased modestly over time reaching 3.09, 3.0, 3.40 and 0.69 DDDs/TID in 2019, respectively.

In the subgroup of ‘antihypertensives’ doxazosin and methyldopa were the leading medicines although their utilization was very low, usually less than one and 0.5 DDDs/TID, respectively, followed by methyldopa with the use less than 0.5 DDDs/TID (Figure 6B). Due to unproven therapeutic effect and increased potential for side effects he fixed dose combination containing reserpine and clopamide with dihydroegocristine was completely withdrawn from the positive medicine list in 2013.

**FIGURE 7:**
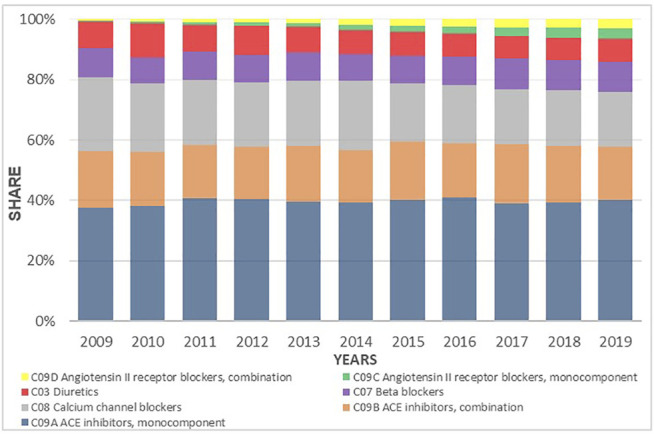
Utilization share of antihypertensive medicines groups and subgroups from 2009 to 2019 expressed in percentages.

Despite the fact that the total prescribing of all classes of antihypertensive medicines increased during the investigated period, the utilization share between the subgroups did not appreciably change. ACEIs as mono therapy or in combination with a diuretic were the leading antihypertensive medicines dispensed during the study period with a 47% share of prescriptions dispensed on a DDDs basis. The CCBs were the second most commonly dispensed although their relative contribution decreased from 24.2% to 18.2%. This was followed by BBs with 10.1% and diuretics with 7.6% of total utilization. The ARBs, both as monotherapy and in combination with HCTZ, remained constant during the period of time, reaching 3.2% of the annual share in 2019 (Figure 7).

The number of antihypertensive medicines in the DU90% segment varied from 16 in 2009 to 18 in 2015 and 19 in 2019. The DU90% segment in 2009 included all antihypertensive subgroups except ARBs (C09C, C09D), and antihypertensives (C02), while in 2015 and 2019 it included all except antihypertensives (C02). The number of fixed dose combinations of ACEI with HCTZ in the DU90% segment increased from two in 2009 to four in 2015 and 2019. No fixed dose combinations of ARB with HCTZ were seen within DU90% segment in 2009, but in 2019 two medicine combinations of this type were present (Table 4).

**TABLE 4:** Antihypertensive medicines utilization expressed in DDDs/TID as DU90% segment in the Republic of Srpska during 3 years, 2009, 2015 and 2019.

No	2009			2015			2019		
	ATC	%	DDD/TID	ATC	%	DDD/TID	ATC	%	DDD/TID
1	C09AA02	27.2	34.27	C09AA02	27.11	80.31	C09AA02	21.99	90.37
2	C08CA01	20.03	25.21	C08CA01	15.48	45.86	C08CA01	12.63	51.92
3	C09BA02	12.33	15.52	C09BA02	13.2	39.09	C09BA02	10.51	43.2
4	C07AB02	4.68	5.9	C09AA05	5.94	17.59	C09AA05	10.08	41.44
5	C03CA01	4.39	5.53	C03CA01	4.57	13.53	C03CA01	4.94	20.3
6	C09BA06	4.03	5.08	C07AB02	4.1	12.12	C07AB07	3.83	15.74
7	C09AA05	2.47	3.11	C09AA03	2.72	8.07	C08CA13	3.77	15.5
8	C09AA06	2.32	2.92	C09BA03	2.66	7.89	C09AA03	3.73	15.34
9	C07AB03	2.15	2.71	C07AB07	2.15	6.38	C07AB02	3.23	13.26
10	C08DA01	1.87	2.36	C09BA06	1.94	5.76	C09BA03	2.67	10.98
11	C09AA03	1.71	2.15	C09CA01	1.9	5.62	C09AA06	1.71	7.04
12	C09AA01	1.69	2.13	C09DA01	1.78	5.27	C09CA01	1.71	7.03
13	C08CA05	1.69	2.13	C09AA06	1.54	4.55	C09DA01	1.7	6.98
14	C07AG02	1.52	1.91	C09AA01	1.32	3.9	C09BA06	1.68	6.91
15	C03AA03	1.18	1.49	C09AA09	1.26	3.74	C07AB12	1.37	5.63
16	C03EA01	1.14	1.44	C09BA05	1.2	3.54	C09BA05	1.61	6.61
17	—	—	—	C03AA03	1.08	3.21	C09CA04	1.26	5.19
18	—	—	—	C08CA13	1.08	3.2	C09AA09	1.2	4.95
19	—	—	—	—	—	—	C09DA04	1.05	4.3
DU90% 1–16 (average)	—	90.4	113.86	—	91.03	269.63	—	90.67	372.69
Others 17–55 (average)	—	9.6	12.14	—	8.97	26.6	—	9.33	38.3
Total 1–55	—	100	126	—	100	296.23	—	100	410.99

HCTZ, hydrochlorothiazide; C09AA02-enalapril; C08CA01-amlodipine; C09BA02-enalapril, HCTZ; C09AA05-ramipril; C03CA01-furosemide; C07AB07-bisoprolol; C08CA13-lercanidipine; C09AA03-lisinopril; C07AB02-metoprolol; C09BA03-lisinopril, HCTZ; C09AA06-quinapril; C09CA01-losartan; C09DA01-losartan, HCTZ; C09BA06-quinapril, HCTZ; C07AB12-nebivolol; C09BA05-ramipril, HCTZ; C09CA04-irbesartan; C09AA09-fosinopril; C09DA04-irbesartan, HCTZ; C07AB03-atenolol; C08DA01-verapamil; C09AA01-captopril; C08CA05-nifedipine; C07AG02-carvedilol; C03AA03-HCTZ; C03EA01-amilorid, HCTZ.

## Discussion

The results of this study showed that during the past 11 years, the utilization of antihypertensive medicine in the Republic of Srpska increased more than three times with similar trend already seen in our earlier study (Markovic-Pekovic et al., 2009). At the same time, the number of hypertensive patients increased more than two-fold. The explanation for this appreciable increase in prevalence rates in such a short period could be partly explained by improved preventive measures advocating the prescribing of antihypertensives and a better data collecting system. However, the potential increase in the number of hypertensive patients cannot be neglected especially with growing elderly populations. This reflects a general increase in the number of patients with hypertension in LMICs in recent years.

The additional reason for the increased use of antihypertensive medicines in this period could be explained by better identification and implementation of national and ESC/ESH guidelines for the treatment of hypertension and coronary heart disease in the Republic of Srpska. It is clearly stated in these guidelines that all antihypertensive medicines are suitable and available for the treatment of hypertension, either as mono- or combination therapy, and that treatment should be initiated at the early stage of the disease (Mancia et al., 2013). Based on that recommendations, all hypertensive patients should be treated with low doses of thiazides or thiazide-like diuretics, and that ACEIs, ARBs, CCBs or BBs should be prescribing taking into consideration the patient’s health status and the presence of any comorbidities (Mancia et al., 2013; Ministry of Health and Social Welfare of Republic of Srpska, 2015). It is important to mention that recommendations in the national guidelines for treatment options and combination therapy in the Republic of Srpska have been fully harmonized with the ESH/ESC guidelines (Zanchetti, 20032003; Guidelines Committee, 2004; Guidelines Committee, 2009; Perk et al., 2012; Ministry of Health and Social Welfare of Republic of Srpska, 2015; Williams et al., 2018).

The trends of antihypertensive medicines use are also influenced by registration status and national drug policy presented by HIF positive medicine lists. Based on the HIF rules, the family practitioners as exclusive prescribers at the primary healthcare level are obliged to prescribe medicines listed on positive medicine list according to INN (Health Insurance Fund of Republic of Srpska, 2021a; Health Insurance Fund of Republic of Srpska, 2021b). The appreciable increase in the utilization of cardiovascular medicines from 163.81 DDD/TID in 2009 to 486.93 DDD/TID in 2019 is similar to the previously published studies form neighboring countries including Croatia and Serbia, as well as other European countries (Tomas et al., 2016; Lisauskienė et al., 2017; Sundbøll et al., 2017; Kucan et al., 2018). The significant usage of ACEIs seen in this study, rising more than three-fold during the study period, was also seen in the DU90% profile–out of 16 medicines within DU90% in 2009, seven were ACEIs and their combinations with diuretics. The number of these antihypertensives within the DU90% was ten of 18 in 2015 and nine of 19 in 2019. However, diuretics, which are recommended as first line therapy are ranked as fourth in total antihypertensive medicine utilization (National Institute for Health and Care Excellence, 2011; Borghi et al., 2020).

We believe this appreciable increase in the prescribing of ACEIs during the study period was fuelled by the HOPE (Yusuf et al., 2000) and EUROPA (Fox et al., 2003) trials, and confirmed by ESH/ESC guidelines (Mancia et al., 20072007) that were adopted by National Cardiology Society of the Republic of Srpska in the national Guidelines for Arterial Hypertension, Second Update from 2009 (Guidelines Committee, 2009). These medicines are primarily used to treat hypertension and congestive heart failure as monotherapy or in combination with diuretics. The fixed-dose combinations also offer the potential to lower blood pressure more quickly with decreased adverse effects (Frank, 2008). It has been clearly emphasized and confirmed that low-dose combinations of antihypertensive agents in general are more effective in blood pressure lowering and with fewer side effects than high-dose monotherapies (Atkins and Chow, 2020).

During 2009 to 2019, the use of ARBs also significantly increased. This was mainly due to the sustained increase in losartan prescribing and further reinforced by adding irbesartan, valsartan and telmisartan on the HIF positive medicine lists following national Guidelines on Secondary Prevention of Coronary Heart Disease from 2011 (Vulić, 2013) and Guidelines for Arterial Hypertension from 2015 (Ministry of Health and Social Welfare of Republic of Srpska, 2015). The ARBs were not present on DU90% profile in 2009, but in 2015 and 2019 this group was presented with two and four INNs, respectively, due to generic availability of these medicines in the country. We believe this steady increase in the prescribing of ARBs was based on favorable results of clinical trials demonstrating beneficial effects of valsartan (Cohn and Tognoni, 2001), losartan (Dahlöf et al., 2002) and candesartan (Young et al., 2004) on various outcomes in patients with hypertension or hearth failure, as well as with better tolerability and less side effects compared to ACEIs.

CCBs were the second most prescribed group of antihypertensive medicines with a 3.5-fold increase in 2019 compared to 2009, with amlodipine accounting for more than 80% of all CCBs in 2009 and 62% in 2019. The favorable effects of amlodipine in reducing major cardiovascular events in patients with hypertension shown in clinical trials, including VALUE (Julius et al., 2004), ACCOT-BPLA (Dahlöf et al., 2005) and ACCOMPLISH (Jamerson et al., 2008), may well have enhanced its use. Moreover, amlodipine as a long-acting medicine, has shown more acceptable safety profile with better patient compliance than previously used nifedipine. The use of lercanidipine significantly increased after it was introduced in the national guidelines and the HIF positive medicine list in 2011 due to its lower incidence of side effects and its availability as a generic medicine.

It has become obvious that most patients in the Republic of Srpska and globally require two or more antihypertensive medicines to achieve blood pressure control. Fixed-dose combinations (FDCs) of ACEIs or ARBs with diuretics or CCBs can help with improved patient compliance compared with multiple medicine administration and improve outcomes (Kawalec et al., 2018; Verma et al., 2018; Webster et al., 2018; Benjamin et al., 2019; Hassanein, 2020). These medicines, mainly ARBs and their combinations with diuretics are widely used in Norway, Finland, Denmark, with well-developed pharmacotherapeutic practices [ (NSDUH, 2014; Norwegian Institute for Public Health, 2014; Sundbøll et al., 2017). Similar trends were observed in studies from Croatia and Serbia (Tomas et al., 2016; Kucan et al., 2018). However, there can be concerns with an increase in medication errors with FDCs in patients with hypertension (Moriarty et al., 2019).

The reason for the increasing use of bisoprolol could be partly explained by the results of CIBIS-II (Dargie and Lechat, 1999) trial which demonstrated a relative risk reduction in all-cause mortality for patients with heart failure. The significant increase in furosemide prescribing during the study period could be due to an increase in the number of patients with comorbidities, particularly heart failure and those with poorly controlled and/or resistant hypertension. The prescribing of indapamide increased more than 8 times during the study period supported by HYVET trial, which provided evidence that antihypertensive treatment with indapamide reduces all-cause mortality in older patients (Kostis, 2008). Both, national and international guidelines clearly stated the superiority and effectiveness of the thiazide diuretics in the treatment of hypertension in the elderly, but their consumption in the Republic of Srpska was unreasonably low (National Institute for Health and Care Excellence, 2011; Williams et al., 2018). This may be due to their well-known side effects and concerns with adherence in this patient population leading to increased prescribing of FDCs. We will be exploring this further in future studies.

Based on our findings, it can be seen that the implementation of guidelines has influenced the prescribing patterns of antihypertensive medicines, especially ACEIs and ARBs with thiazides, during the study period. The main changes in the utilization patterns seen strongly correlated with the recommendations proposed by national and international guidelines for hypertension, for instance in 2015, where CCBs, ACEIs and ARBs as monotherapy were switched to combination therapy with diuretics. However, the findings regarding the utilization of diuretics showed that some factors, other than clinical guidelines, including personal or previous experiences have influenced physicians prescribing habits. Alongside this medicine promotion, media coverage, market competition and purchasing contracts may influence physician prescribing practices; however, medicines outside the positive list are subject to high patient co-payment levels impacting on their influence in practice. It is clear that treatment guidelines has improved doctors prescribing practices, not only in having the better control of patients with hypertension, but also in providing them with most appropriate treatment options according to the specific patient’s needs and comorbidities. However, in this study we did not deal with treatment outcomes, and at this moment it would be difficult to prove that implementation of clinical guidelines has been associated with significant changes in major adverse cardiovascular events.

In addition to guidelines on treating hypertension that are widely available, it is becoming more important to perform pharmacoepidemilogic analyses that might reveal the trends of the most frequently used medicine groups as initial therapy. Subsequently, based on the findings, it would be reasonable to redefine treatment strategies in order to improve prescribing habits especially if this revolves around new national robust treatment guidelines.

## Strengths and Limitations

Pharmacoepidemiological analyses serve as an important tool for health professionals to track down medicinal expenditures over the period of time and to improve their prescribing skills, which is important on this occasion to help reach therapeutic goals in hypertension treatment.

This study has several limitations. First, it was unable to link the medicine utilization database to patient’s diagnoses including the number of patients with comorbidities that could influence the medicine utilization figures. Second, this study cannot account for the actual use of antihypertensive medications since treatment adherence has always been a problem in the management of hypertension. However, it is unlikely that these factors could significantly influence the overall figures of utilization of antihypertensive medicines in observed period.

## Conclusion

The present study showed an appreciable increase in antihypertensive medicine utilization in the Republic of Srpska between 2009 and 2019. The overall increase was driven by increased prescribing of ACEIs (enalapril, ramipril, lisinopril, both as monotherapy and in combination with thiazides), BBs (metoprolol, bisoprolol, nebivolol), CCBs (amlodipine, lercanidipine), ARBs (losartan, irbesartan, both as monotherapy and in combination with thiazides) and diuretics (furosemide). These results are comparable with results from other countries. Except for diuretics, the overall antihypertensive medicine utilization was largely influenced with national and ESH/ESC guidelines for hypertension.

## Data Availability

The original contributions presented in the study are included in the article/supplementary material, further inquiries can be directed to the corresponding author.
